# Activation of miR-34a-5p/Sirt1/p66shc pathway contributes to doxorubicin-induced cardiotoxicity

**DOI:** 10.1038/s41598-017-12192-y

**Published:** 2017-09-19

**Authors:** Jie-Ning Zhu, Yong-Heng Fu, Zhi-qin Hu, Wen-Yu Li, Chun-Mei Tang, Hong-Wen Fei, Hui Yang, Qiu-xiong Lin, De-Ming Gou, Shu-Lin Wu, Zhi-Xin Shan

**Affiliations:** 1 Guangdong Cardiovascular Institute, Guangdong Provincial Key Laboratory of Clinical Pharmacology, Guangzhou, 510080 China; 2 Research Center of Medical Sciences, Guangdong General Hospital, Guangdong Academy of Medical Sciences, Guangzhou, 510080 China; 3 Lymphoma Division, Cancer Center, Guangdong General Hospital, Guangdong Academy of Medical Sciences, Guangzhou, 510080 China; 4 College of Life Science, Shenzhen University, Shenzhen, Guangdong, 518060 China

## Abstract

The molecular mechanisms underlying anthracyclines-induced cardiotoxicity have not been well elucidated. MiRNAs were revealed dysregulated in the myocardium and plasma of rats received Dox treatment. MicroRNA-34a-5p (miR-34a-5p) was verified increased in the myocardium and plasma of Dox-treated rats, but was reversed in rats received Dox plus DEX treatments. Human miR-34a-5p was also observed increased in the plasma of patients with diffuse large B-cell lymphoma after 9- and 16-week epirubicin therapy. Up-regulation of miR-34a-5p was observed in Dox-induced rat cardiomyocyte H9c2 cells. MiR-34a-5p could augment Bax expression, but inhibited Bcl-2 expression, along with the increases of the activated caspase-3 and mitochondrial potentials in H9C2 cells. MiR-34a-5p was verified to modulate Sirt1 expression post-transcriptionally. In parallel to Sirt1 siRNA, miR-34a-5p could enhance p66shc expression, accompanied by increases of Bax and the activated caspase-3 and a decrease of Bcl-2 in H9c2 cells. Moreover, enforced expression of Sirt1 alleviated Dox-induced apoptosis of H9c2 cells, with suppressing levels of p66shc, Bax, the activated caspase-3 and miR-34a-5p, and enhancing Bcl-2 expression. Therefore, miR-34a-5p enhances cardiomyocyte apoptosis by targeting Sirt1, activation of miR-34a-5p/Sirt1/p66shc pathway contributes to Dox-induced cardiotoxicity, and blockage of this pathway represents a potential cardioprotective effect against anthracyclines.

## Introduction

Anthracyclines, used alone or in combination with other chemotherapeutic agents, are a class of antitumour drugs widely used for the treatment of a variety of cancers. The commonly used anthracyclines include doxorubicin (Dox), daunorubicin, and epirubicin^1^. Dox is one of the most widely used and successful antitumor drugs, but its cumulative and dose-dependent cardiotoxicity limits its clinical application^2^. The reactive oxygen species (ROS) have been recognized to participate in Dox-induced cardiotoxicity^3^, and iron may catalyze ROS formation contributing to Dox-induced cardiotoxicity^4,5^. However, the mechanisms underlying Dox-induced cardiotoxicity remain elusive. Dexrazoxane (DEX) has been approved to prevent anthracycline-induced cardiomyopathy^6,7^. The co-administration of DEX with each dose of anthracycline has been reported to efficiently attenuate cardiotoxicity^8–10^. DEX is thought to chelate free iron to prevent the generation of free radicals that lead to oxidative damage to cardiomycytes^11^.

MicroRNAs (miRNAs) are endogenous, non-coding, 20–23 nucleotide RNAs that negatively regulate a variety of target genes. Increasing evidences have correlated dysregulated miRNA expression to cardiovascular diseases^12–15^. Recent studies have demonstrated that miRNAs participate in Dox-induced cardiomyopathy^16,17^. However, the potential roles of miRNAs in Dox-induced cardiotoxicity are not well illustrated.

In this study, we investigated the achievement of cardiac remodeling, expressions of apoptosis-related genes and miRNA profiling in the myocardium of SD rats received Dox or Dox combined with DEX treatment. MiR-34a-5p was identified consistently increased in the myocardium and plasma of Dox-treated rats, and plasma hsa-miR-34a-5p was also confirmed increased in lymphoma patients received Dox therapy. Sirt1 was identified as a target gene of miR-34a-5p in Dox-induced cardiotoxicity. MiR-34a-5p could augment p66shc expression through suppressing Sirt1 to result in increased expression of Bax, activated caspase-3 and decreased expression of Bcl-2, contributing to Dox-induced apoptosis of cardiomyocytes. Our data demonstrated the NF-κB signal pathway mediates the upregulation of miR-34a-5p in cardiomyocytes exposed to Dox treatment. Overall, we identified miR-34a-5p/Sirt1/p66shc pathway mediates doxorubicin-induced apoptosis of cardiomyocytes and possibly provided a valuable way to protect against Dox-induced cardiotoxicity.

## Methods

### Patients

A total of 22 adult patients with high-risk diffuse large B-cell lymphoma (DLBCL) between July 2013 and February 2014 in Guangdong General Hospital, China were enrolled in this study. Positive human immunodeficiency virus (HIV) status and pregnancy were exclusive criteria. All patients received a dose of 75 mg/m^2^ epirubicin (a kind of Dox) i.v. every 3 weeks for 6 times. Two mL peripheral blood was collected for detection of concerned plasma miRNAs at 3 time points, including before initiating treatment, 9 weeks and 16 weeks after initiating treatment. This study protocol was performed conform the declaration of Helsinki and has been approved by the ethics committees of Guangdong General Hospital. All patients provided informed consent to allow the use of their blood samples for research purposes.

### Animals

Male sprague-dawley (SD) rats weighing 250 ± 9 g, license number SCXK (YUE) 2004–0011 (Department of Experimental Animal Research Center, Sun Yat-sen Medical College, Sun Yat-sen University, Guangzhou, China) were used. All animals were housed under pathogen-free conditions and kept on standard mouse chow with free access to tap water. This study conformed to the Guide for the Care and Use of Laboratory Animals published by the US National Institutes of Health (8th Edition, National Research Council, 2011). All methods and experimental protocols in the present program were also approved by the research ethics committee of Guangdong General Hospital (the approval number: No. GDREC2010093A). In the present study, SD rats received Dox intravenously (IV) via tail vein injections twice a week for 4, or 8 weeks at a dose of 2 mg/kg (cumulative dose: 16 mg/kg or 32 mg/kg) to achieve cardiotoxicity. Animals in the Dox + DEX groups were given an intraperitoneal (IP) injection of 20 mg/kg DEX followed (30 minutes later) by an IV injection of 2 mg/kg DOX twice a week for 4, or 8 weeks.

### Echocardiographic study

Left ventricular (LV) function variables were assessed by transthoracic echocardiography. After the induction of light general anesthesia, the rats underwent transthoracic twodimensional (2D) guided M-mode echocardiography with an 8.5-MHz transducer (Acuson, Mountain View, CA). From the cardiac short axis (papillary level), the LV anterior wall enddiastolic thickness (LVAWd), the systolic LV anterior wall thickness(LVAWs), the LV internal dimension at end-diastole (LVIDd), the LV internal dimension at end-systole(LVIDs), the LV posterior wall end-diastolic thickness (LVPWd), the LV posterior wall end-systolic thickness(LVPWs), the ejection fraction (EF) and fractional shortening (FS) were measured. Echocardiographic measurements were averaged from at least three separate cardiac cycles.

### Detection of plasma cTnT, MDA and Ca-Mg-ATP enzyme activity

Levels of plasma cTnT and MDA, and Ca-Mg-ATP enzyme activity were detected according to the protocols of the corresponding assay kit instructions (Nanjing Jiancheng Bioengineering Institute, Nanjing, China).

### Histological analysis

Rats were sacrificed with an intraperitoneal injection of 2 mL of pentobarbital. The heart was excised, and the LV myocardium fixed overnight in 10% formalin. Samples were embedded in paraffin and cut into 4 μm thick sections. They were mounted on normal glass slides and stained with Masson trichrome for histological examination. For the collagen volume fraction (CVF) analysis, eight separate views (magnification = original × 400) were selected and assessment of CVF used the following formula: CVF = collagen area/total area.

### miRNA microarray

MiRNA expression analysis was performed on total RNA extracted from a pool of 5 to 8 myocardium or plasma samples of rats received Dox or Dox + DEX treatment by using Trizol reagent and Trizol LS reagent (Invitrogen, Carlsbad, CA, USA), respectively. Microarray procedures and data analysis were performed at Bioassay Laboratory of CapitalBio Corporation (Beijing, China). Briefly, 200 ng of total RNA extracted from myocardium or plasma samples was fluorescently labeled with Cyanine3-pCp using miRNA Complete Labeling and Hyb Kit (Agilent Technologies, Santa Clara, CA, USA). The labeled samples were then concentrated and hybridized with the Hybridization Chamber gasket slides (Agilent, USA). Arrays were scanned on Agilent chip scanner (G2565CA), and image analysis was performed using Agilent Feature Extraction(v10.7) software (Agilent, USA), followed with data normalization using Agilent GeneSpring software (Agilent, USA).

### Cell culture and treatments

Rat cardiomyocyte H9c2 cells were grown to confluency on 10-cm cell culture dishes in growth media (DMEM/LG 10% FBS, 1% penicillin and 1% streptomycin) at 37 °C in humid air with 5% CO_2_. H9c2 cells were incubated with 1 to 5 μM Dox for 24 h to induce the apoptotic phenotype. Cells were treated with NF-κB inhibitor JSH23 (10 μM) or QNZ (5 nM). Cells were transfected with 50 nM scramble, miR-34a-5p mimic, Sirt1 siRNA and NF-κBP65 by oligofectamine reagent (Invitrogen, Carlsbad, CA), and were transfected with pcDNA3-Sirt1 by lipofectamine 2000 reagent (Invitrogen, CA).

### Terminal deoxynucleotidyl transferase dUTP nick end labeling (TUNEL) assay

Terminal deoxynucleotidyl transferase dUTP nick end labeling (TUNEL) assay was performed as in our previous report^18^. Briefly, H9c2 cells were cultured on coverslips and were fixed in 4% paraformaldehyde after experimental treatments, followed with permeabilization with 0.2% Triton X-100. Cy5-dUTP (Amersham, Piscataway, NJ, USA) was used to label DNA strand breaks in the apoptotic cells. The level of TUNEL-positive cells was detected by fluorescence microscopy, about 200 cells per field in five different visual fields were counted in the present study.

### Determination of mitochondrial membrane potential (ΔΨm)

At 24 h post-transfection of miR-34a-5p mimic or scramble control, H9c2 cells were washed thrice with PBS (pH 7.2, 1 mL), then incubated for 15 min with 3 μM rhodamine 123 (Molecular Probes, USA) in PBS. Cell suspensions were incubated for 15 min at 37 °C. Cells were subsequently analyzed with a flow cytometer (Beckman, USA). Results were expressed as the proportion of cells exhibiting low mitochondrial membrane potential estimated by reduced uptake of rhodamine 123.

### Quantitative mRNA and miRNA measurements

For detection of mRNA expression of coding genes, first-strand cDNA was generated from 1.5 μg total RNA using a mixture of oligo (dT)_15_ and random primers with superscript reverse transcriptase (Invitrogen, Carlsbad, CA). Expression of miR-34a-3p in rat myocardium and H9c2 cells was detected by RT-qPCR as previous report^19^. Determination of miR-34a-5p in the supernatant of H9c2 cells or in the plasma of rats and DLBCL patients were performed by using the poly(A) method^20^ as follows. In brief, 300 μl cell supernatant or plasma was incubated with 1 ml Trizol LS reagent (Invitrogen), followed with total RNA extraction with 200 μl phenol/chloroform. Ten μl RNase-free water was used to re-suspend the RNA pellet, and 4 μl RNA solution could be used for one cDNA synthesis reaction. After polyadenylation, the reverse transcription of miRNA was carried out using a universal RT primer that contains degenerate nucleotides at 3′ end followed by an oligo(dT) and universal reverse primer sequence. The cDNA was amplified with specific forward and universal reverse primers. To normalize RNA content, the exogenous cel-miR-54 was used for plasma miR-34a-5p template normalization. The amplification products were detected by SYBR Green I. PCR and analyses were performed with a ViiA7 Quantitative PCR System (Applied Biosystems, Carlsbad, CA). The 2^−∆∆Ct^ method was used to calculate relative expression levels of coding genes and miR-34a-5p between treatments. PCR primers for coding genes are shown in Supplementary Table 1.

### Western blot analysis

The amount of 40 μg protein prepared from rat myocardium or H9c2 cells was used in a standard Western blot analysis. The polyvinylidene fluoride (PVDF) membrane binding sample protein was incubated with a high affinity anti-Bax antibody (1:2000 dilution), anti-Bcl-2 antibody (1:1000) (Abcam, Cambridge, MA), anti-caspase-3 antibody (1:2000), anti-Sirt1 antibody (1:1000) and anti-p66shc antibody (1:2000) (Cell Signaling Technology, Danvers, MA), respectively. An anti-GAPDH antibody (1:2000) (Santa Cruz Biotechnology, Santa Cruz, CA) was used to detect level of GAPDH as an internal control. Proteins were visualized using the ECL Plus detection system (GE Healthcare, Waukesha, WI).

### Dual luciferase assay for Sirt1 target identification

As in our previous report^21^, the recombinant luciferase reporter plasmids containing sequences of potential miR-34a-5p binding sites in 3ʹ UTR of Sirt1 gene were constructed. Using a site-directed mutagenesis kit (TransGen, Beijing, China), miR-34a-5p complementary binding sequence ACUGCC was replaced with AGACGC to construct recombinant luciferase reporter plasmids containing the mutant potential miR-34a-5p binding sequences.

Human embryonic kidney (HEK) 293 cells (3 × 10^5^ cells per well in 12-well plate) were co-transfected with 200 ng of recombinant luciferase reporter plasmid, 50 nM miR-34a-5p mimic, and 20 ng of pRL-TK as an internal control (Promega, Madison, WI). Activities of firefly luciferase (FL) and Renilla luciferase (RL) were measured 24 hr after transfection, and the relative ratio of the FL/RL was used to indicate the miR-34a-5p-mediated knockdown of Sirt1.

### Statistical analysis

The data are presented as the means ± s.e.m. In each experiment, all determinations were performed at least in triplicate. Statistical significance between two measurements was determined by the two tailed unpaired Student’s *t* test, and among groups, it was determined by one-way ANOVA. A value of *p* < 0.05 was considered to be significant.

## Results

### MiR-34a-5p was upregulated in the myocardium of Dox-treated rats

**Figure 1:**
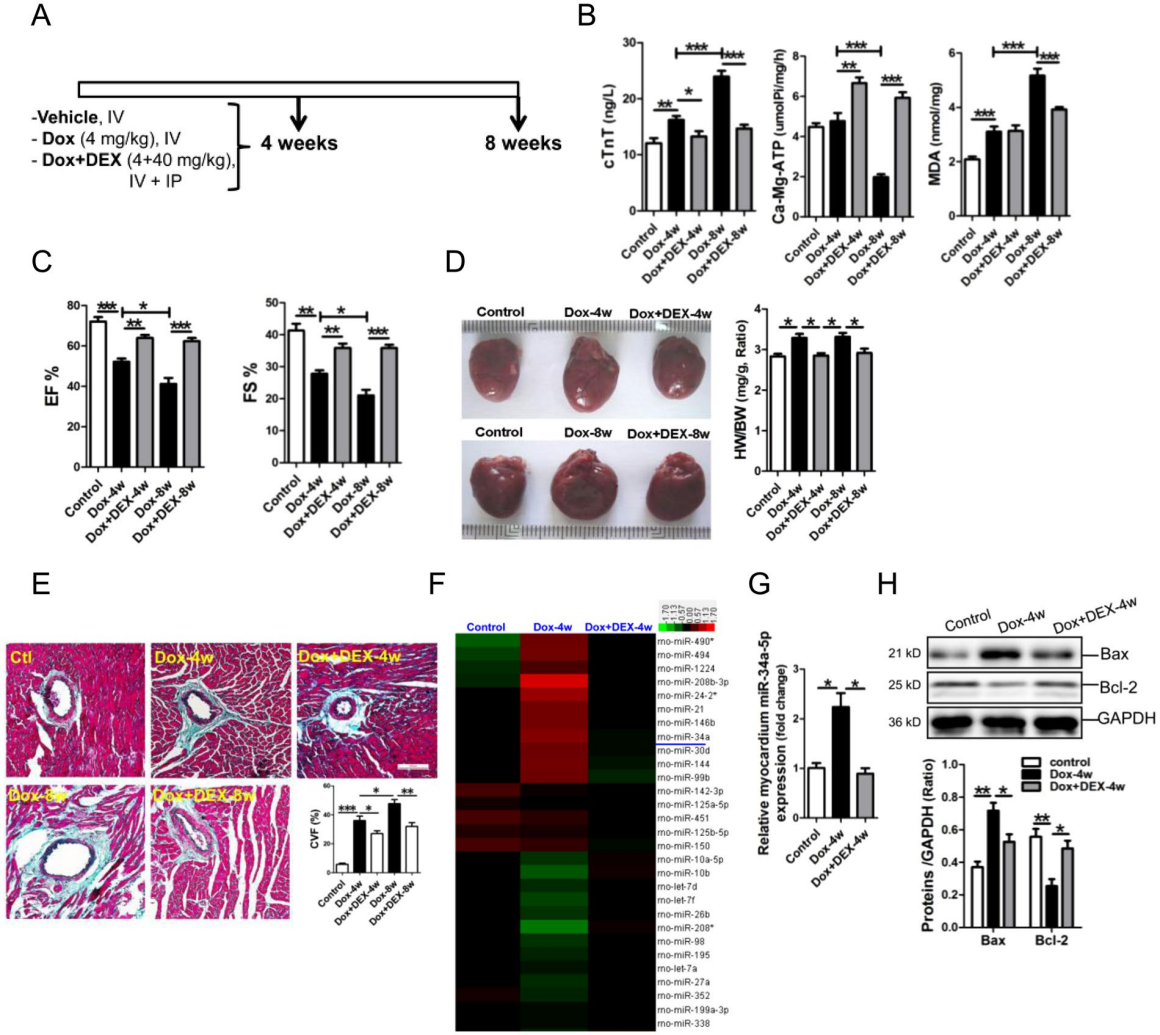
miR-34a-5p was upregulated in the myocardium of rats received Dox treatment. (**A**) Schematic outline of rats received Dox and DEX treatments and study time-points. (**B**) Determinations of plasma MDA, cTnT and Ca-Mg-ATP in rats. (**C**) Assessment of rat cardiac function by echocardiography. (**D**) Morphology of rat hearts in each group. (**E**) Masson trichrome stainning. The scale bar was 100 μm. (**F**) Heatmap of dysregulated miRNAs over 1.5 fold in rat myocardium. (**G**) Expression of miR-34a-5p in rat myocardium by RT-qPCR assay. (**H**) Protein expression of Bax and Bcl-2 in rat myocardium by Western blot assay. Data are shown as mean ± sem, *p < 0.05, **p < 0.01, ***p < 0.001. *n* = 5–8.

Male rats were treated with Dox according to the scheme shown in Fig. 1A. The plasma cardiac troponin T (cTnT) was observed increased at 4 weeks and 8 weeks post-Dox treatment, but DEX could efficiently alleviate the increase of plasma cTnT (Fig. 1B). Activity of plasma Ca-Mg-ATP enzyme was shown decreased in rats after 8-week Dox treatment, but was significantly increased in rats after 4 or 8-week treatment of Dox combined with DEX (Fig. 1B). Significant increase of rat plasma malondialdilyde (MDA) was observed after 4 or 8-week Dox treatment, but the increased plasma MDA was reversed in rats received 8-week treatment of Dox plus DEX (Fig. 1B).

Echocardiography was performed to reveal cardiac structure and function changes in Dox-treated rats with or without DEX injection. Significant decreases of LVAWs and LVPWs, and significant increases of LVIDd and LVIDs were shown after 4 or 8-week Dox treatment, and significant decreases of LVAWd and LVPWd were also oberserved after 8-week Dox treatment. Compared with 4-week Dox treatment, increase of LVIDs was significantly attenuated in rats received 4-week Dox plus DEX treatments. In addition, compared with 8-week Dox treatment, an increase of LVIDs and decreases of LVAWd, LVAWs and LVPws could be markedly reversed in rats received 8-week Dox plus DEX treatments (Supplementary Table 2). In addition, DEX injection could efficiently reverse the decreases of cardiac ejection fraction (EF) and fractional shortening (FS) in rats after 4 or 8-week Dox treatment (Fig. 1C).

The hearts were shown significantly expanded in rats received 4 or 8-week Dox treatment, but not in rats received 4 or 8-week Dox combined with DEX injection (Fig. 1D). Masson staining results revealed that the myocardial perivascular fibrosis was markedly increased in Dox-treated rats, but which could be reversed by DEX treatment (Fig. 1E) (*p* < 0.05, *p* < 0.01, respectively).

MicroRNA microarray was performed to explore the dysregulated microRNAs involved in Dox-induced toxic cardiomyopathy. Consistent with the Heatmap analysis of the dysregulated miRNAs over 1.5 fold in rat myocardium, results of RT-qPCR assay confirmed that miR-34a-5p was significantly upregulated in the myocardium of 4-week Dox-treated rats, but were reversed in the myocardium of rats received 4-week Dox plus DEX treatments (Fig. 1F,G). Additionally, expression of the pro-apoptotic Bax was markedly increased in the myocardium of 4-week Dox-treated rats, but was reversed in the myocardium of rats received 4-week Dox plus DEX treatments (Fig. 1G). On the contrary, expression of anti-apoptotic Bcl-2 was significantly decreased in the myocardium of 4-week Dox-treated rats, but was reversed in the myocardium of rats received 4-week Dox plus DEX treatments (Fig. 1G).

### Plasma miR-34a-5p was elevated post-anthracyclines treatment

**Figure 2:**
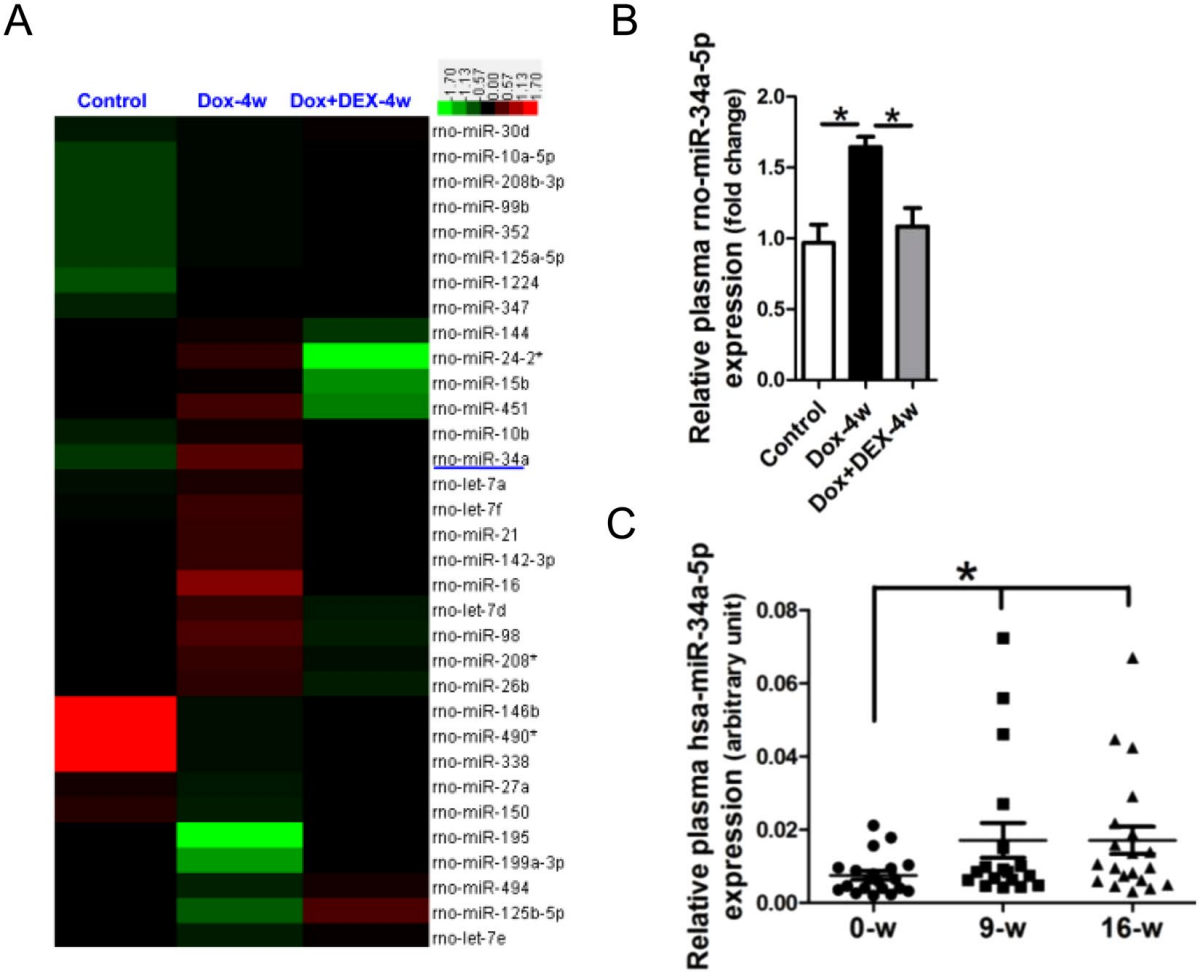
The plasma miR-34a-5p was increased in rats and patients received Dox treatment. (**A**) Heatmap of dysregulated miRNAs over 1.5 fold in rat plasma. (**B**) Determinations of rat plasma miR-34a-5p by RT-qPCR assay. (**C**) Determinations of plasma miR-34a-5p in patients with DLBCL by RT-qPCR assay. Data are shown as mean ± sem, *p < 0.05. *n* = 5–8 for rats, and *n* = 22 for patients.

To investigate the consistently expressed miRNAs in the myocardium and plasma of rats received Dox or Dox plus DEX treatments, miRNAs profiling in rat plasma was also performed in the present study. Consistent with the Heatmap analysis of the dysregulated miRNAs over 1.5 fold in rat plasma, results of RT-qPCR assay confirmed that plasma miR-34a-5p was significantly upregulated in the 4-week Dox-treated rats, but was reversed in rats received 4-week Dox plus DEX treatments (Fig. 2A,B). Levels of plasma hsa-miR-34a-5p was also determined in DLBCL patients received continual 9 and 16-week epirubicin therapy. Results of RT-qPCR assay showed that plasma hsa-miR-34a-5p was significantly increased in DLBCL patients at 9 and 16 weeks after the initiating epirubicin treatment (Fig. 2C).

### MiR-34a-5p participates in Dox-induced apoptosis of rat cardiomyocyte H9C2 cells

**Figure 3:**
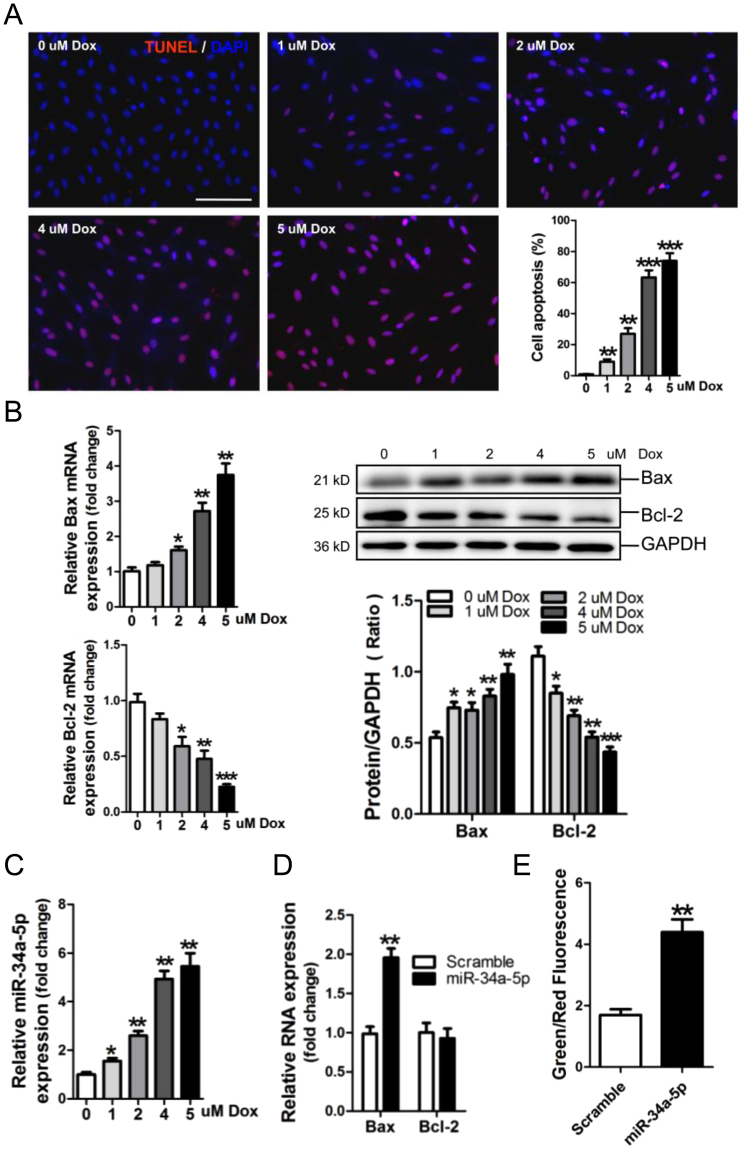
MiR-34a-5p was up-regulated in Dox-treated H9C2 cells. (**A**) The apoptotic H9C2 cells was detected by TUNEL assay. Scale bar is 100 um. (**B**) MRNA and protein expression of Bax and Bcl-2 in H9C2 cells by RT-qPCR and Western-blot assay, respectively. Data are shown as mean ± sem, *p < 0.05, **p < 0.01, ***p < 0.001 vs blank control. *n* = 3. Determination of miR-34a-5p (**C**), Bax and Bcl-2 mRNA expression (**D**) in H9C2 cells by RT-qPCR assay. (**E**) Quantitative analysis of the shifts of mitochondrial potentials in H9C2 cells with transfection of miR-34a-5p mimic. An increase in the bar indicates a shift in the fluorescence ratio correlating with an increase in mitochondrial depolarization. Data are shown as mean ± sem, **p < 0.01 vs scramble control. *n* = 3.

A dose-course study of the effect of Dox on apoptosis of H9c2 cells, and apoptosis-associated Bax and Bcl-2 expressions in H9c2 cells was performed in this study. TUNEL assay showed that 1 to 5 μM Dox could significantly promote apoptosis of H9c2 cells, and 4, 5 μM Dox could induce higher level of apoptosis compared with 1, 2 μM Dox (Fig. 3A). Results of RT-qPCR and Western-blot assay demonstrated that 2, 4 and 5 μM Dox enhance Bax expression, but suppressed Bcl-2 expression at both the mRNA and protein level (Fig. 3B). Meanwhile, miR-34a-5p was observed significantly increased in H9c2 cells exposed to Dox treatment, as well as in the supernatant (Fig. 3C, Supplementary Figure 1). Overexpression of miR-34a-5p could markedly increase expression of Bax mRNA, but not Bcl-2 mRNA, in H9c2 cells (Fig. 3D). The mitochondrial potential assay indicated that the mitochondrial depolarization was significantly increased in miR-34a-5p-modified H9c2 cells (Fig. 3E).

### Identification of Sirt1 as a target of miR-34a-5p

**Figure 4:**
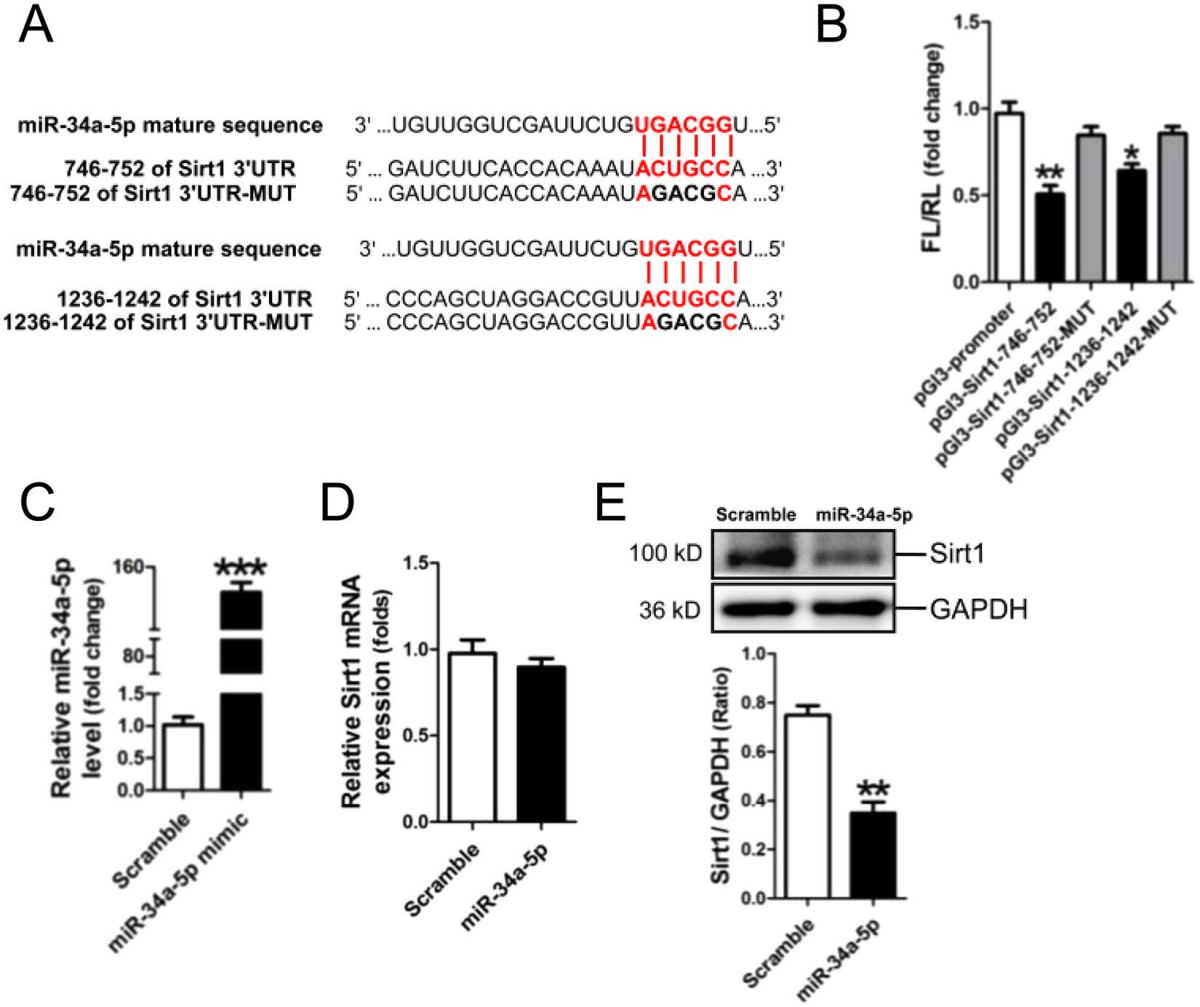
Identification of Sirt1 as a target of miR-34a-5p. (**A**) Predicted miR-34a-5p seed matches to the sequence in the 3ʹUTR of Sirt1 mRNA. The seed sequence of miR-34a-5p is CCGUCA, and the complementary nucleotide sequences are shown in red words. (**B**) Verification of Sirt1 as a target gene of miR-34a-5p by the dual luciferase reporter assay. Data are shown as mean ± sem. *P < 0.05, **P < 0.01 *vs* pGL3-promoter group, *n* = 3. (**C**) Determination of miR-34a-5p in miR-34a-5p-modified H9c2 cells by RT-qPCR assay. Sirt1 mRNA expression (**D**) and protein expression (**E**) in miR-34a-5p-modified H9C2 RT-qPCR and Western blot assay, respectively. Data are shown as mean ± sem. **P < 0.01, ***P < 0.001 vs scramble control, *n* = 3.

Analysis of the databases Mirdb (www.mirdb.org) and TargetScan-Vert (www.targetscan.org) showed that Sirt1was a potential target gene of miR-34a-5p. Two matching positions for miR-34a-5p within 3′-UTR of Sirt1 are shown in Fig. 4A. The dual luciferase assay demonstrated that miR-34a-5p significantly reduced the luciferase activities through binding the following sites, including 764–752, 1236–1242 of 3′-UTR of Sirt1 (*p* < 0.05, *p* < 0.01, respectively) (Fig. 4B).

We examined the expression of Sirt1 in H9c2 cells transfected with miR-34a-5p mimic. RT-qPCR result showed that miR-34a-5p was efficiently transfected into H9c2 cells (*p* < 0.001) (Fig. 4C). Compared with the negative scramble control, Sirt1 protein expression was significantly decreased in miR-34a-5p-modified H9c2 cells (*p* < 0.01), without significant change of Sirt1 mRNA expression (Fig. 4D,E).

### Sirt1/p66shc pathway mediates the pro-apoptotic effect of miR-34a-5p on cardiomyocyres

**Figure 5:**
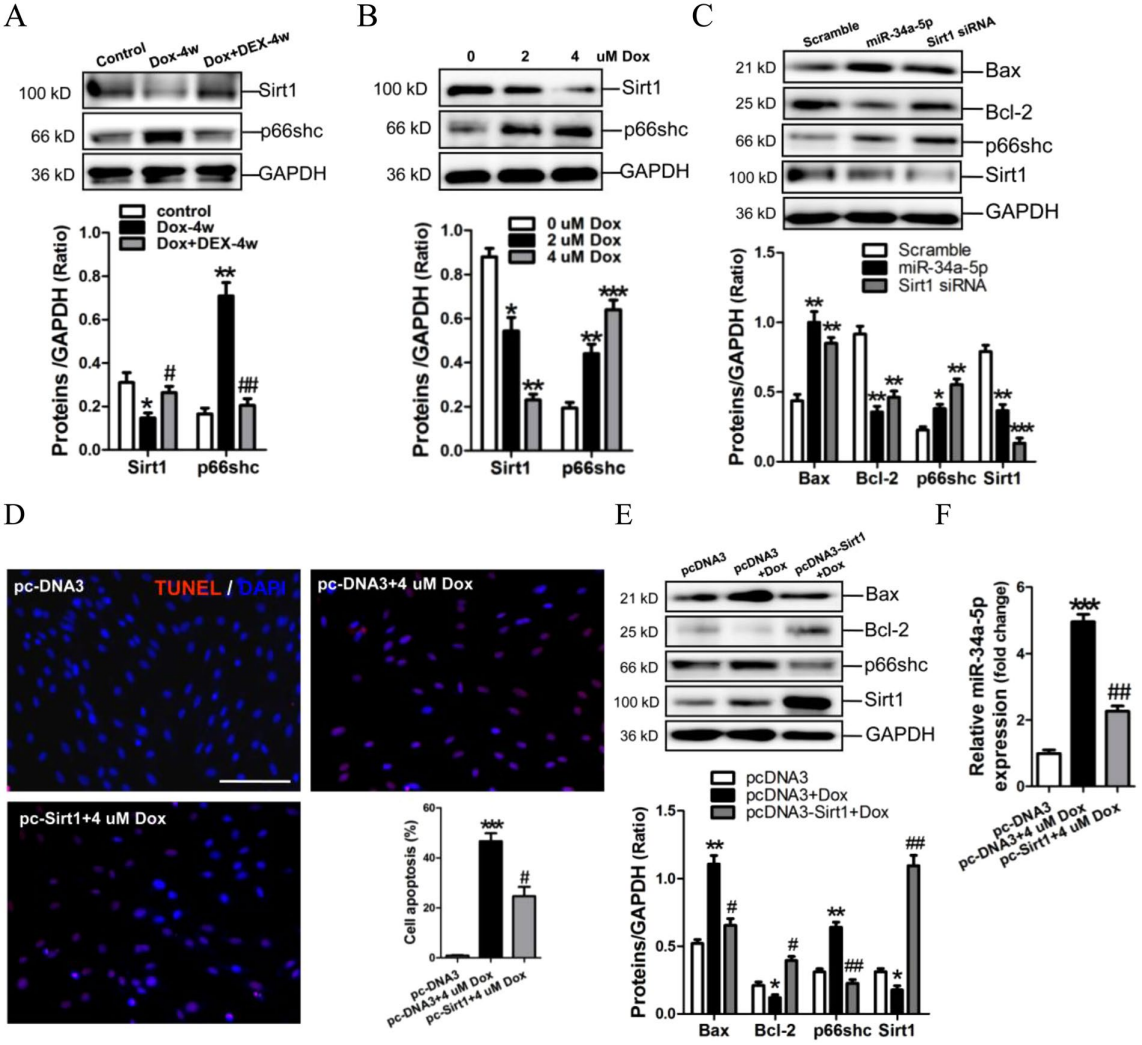
Sirt1 mediates the pro-apoptosis effect of miR-34a-5p in Dox-treated H9C2 cells. (**A**) Expressions of Sirt1 and p66shc in rat myocardium by Western blot assay. Data are shown as mean ± sem. ^*^ *P* < 0.05, ^**^ *P* < 0.01 vs Control group, ^*#*^ *P* < 0.05, ^*##*^ *P* < 0.01 vs Dox-4w group. *n* = 5–8. (**B**) Expressions of Sirt1 and p66shc in H9c2 cells by Western blot assay. Data are shown as mean ± sem. ^*^ *P* < 0.05, ^**^ *P* < 0.01,^***^ *P* < 0.001 vs 0 uM Dox, *n* = 3. (**C**) The protein expression of Bax, Bcl2, p66shc and Sirt1 was determined in H9C2 cells by Western blot assay. Data are shown as mean ± sem. ^*^ *P* < 0.05, ^**^ *P* < 0.01,^***^ *P* < 0.001 vs scramble control group, *n* = 3. (**D**) The apoptosis of H9C2 cells was detected by TUNEL assay. Scale bar is 100 um. (**E**) The protein expression of Bax, Bcl2, p66shc and Sirt1 was determined in H9C2 cells by Western blot assay. (**F**) miR-34a-5p expression in H9C2 cells by RT-qPCR assay. Data are shown as mean ± sem. ^*^ *P* < 0.05, ^**^ *P* < 0.01 vs pcDNA3, ^*#*^ *P* < 0.05, ^*##*^ *P* < 0.01 vs pcDNA3 + Dox. *n* = 3.

P66shc is a target gene of Sirt1, and p66shc promotes mitochondrial death pathway-induced cell apoptosis^22^. Expressions of Sirt1 and p66shc were determined in rat myocardium and H9c2 cells after Dox treatment in the present study. Results of Western blot assay showed that Sirt1 was decreased in the myocardium of Dox-treated rats and was reversed after DEX intervention, contrarily, p66shc was shown increased in myocardium of Dox-treated rats and was alleviated after DEX treatment (Fig. 5A). P66shc protein expression was markedly increased in 2, 4 μM Dox-induced H9c2 cells, with an obvious decrease of Sirt1 expression (*p* < 0.05, *p* < 0.01, *p* < 0.001, respectively) (Fig. 5B). MiR-34a-5p mimic and Sirt1 siRNA were transfected into H9c2 cells. Results of Western-blotting showed that miR-34a-5p mimic and Sirt1 siRNA could efficiently suppress the protein expression of Sirt1, but enhance p66shc expression, along with increases of Bax and the activated caspase-3, and a decrease of Bcl-2 (Fig. 5C, Supplementary Figure 2A).

To further confirm the effect of Sirt1/p66shc pathway in Dox-induced apoptosis of H9c2 cells. Sirt1 was overexpressed in Dox-treated H9c2 cells mediated by pcDNA3 vector. Results of TUNEL assay showed that 4 μM Dox could significantly promote apoptosis of H9c2 cells, and overexpression of Sirt1 could efficiently inhibit Dox-induced cell apoptosis (Fig. 5D). Consistently, Western blotting revealed that enforced expression of Sirt1 could markedly decrease the level of p66shc in H9c2 cells, accompanied with a significant increase of Bcl-2 and decreases of Bax and the activated caspase-3(Fig. 5E, Supplementary Figure 2B). Meanwhile, results of RT-qPCR assay demonstrated that miR-34a-5p was significantly up-regulated by Dox, but could be reversed after over-expression of Sirt1 (Fig. 5F).

### MiR-34a-5p is up-regulated by Dox through the NF-κB pathway in cardiomyocytes

**Figure 6:**
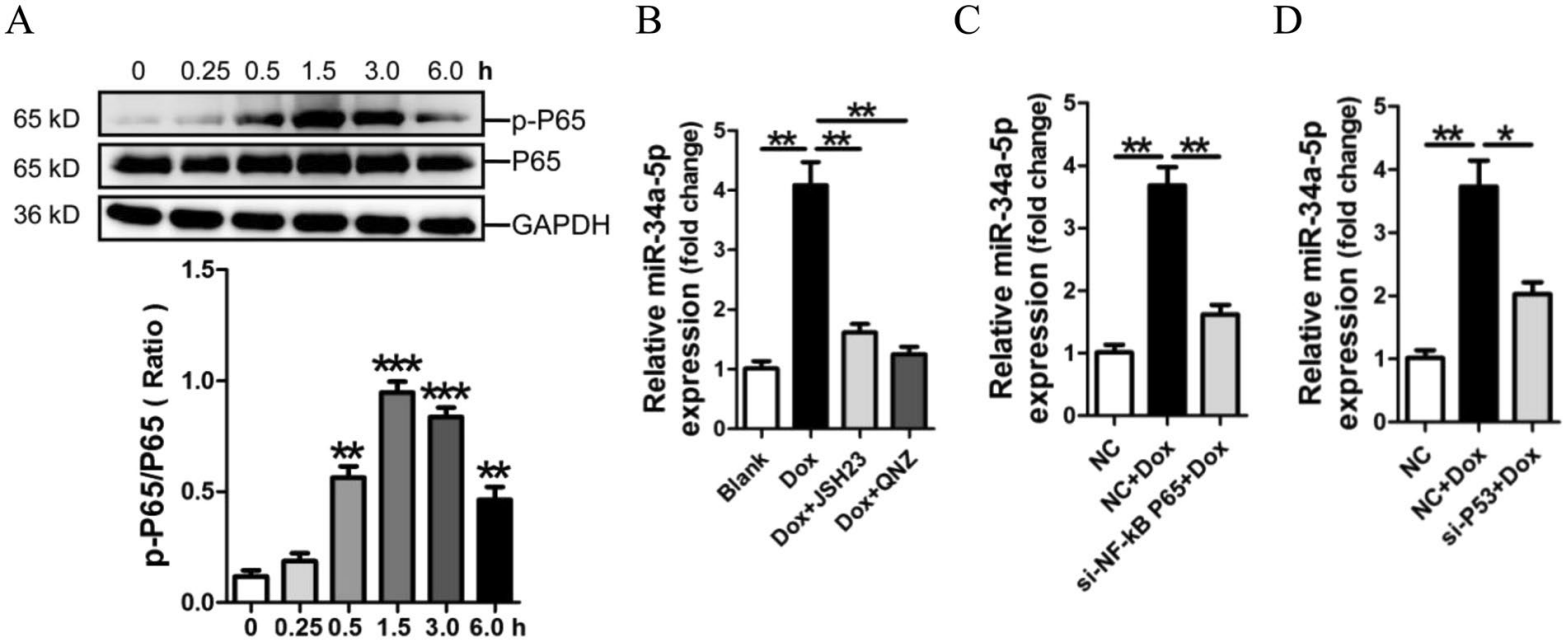
Up-regulation of miR-34a-5p in H9c2 cells through NF-κB pathway. Activation of NF-κB signaling in Dox-treated H9c2 cells in a time-course study (**A**). MiR-34a-5p expression in Dox-induced H9c2 cells with separate pre-treatment with NF-κB inhibitor JSH23 and QNZ (**B**), or with knockdown of P65 (**C**) and P53 (**D**), was assessed by RT-qPCR assay. Data are shown as mean ± sem, ^**^ *P* < 0.01. *n* = 3.

In the current study, we investigated whether the NF-κB pathway mediate the modulation of miR-34a-5p in H9c2 cells. The result of Western blotting showed that the phosphorylation level of NF-κB P65 was significantly increased from 0.5 h to 6 h in H9c2 cells in response to 4 μM Dox treatment (Fig. 6A). We pre-treated H9c2 cells with NF-κB P65 inhibitor JSH23 or QNZ for 0.5 h before 3-hour 4 μM Dox treatment. The RT-qPCR result demonstrated that treatment with either JSH23 or QNZ prevented Dox-induced miR-34a-5p expression (Fig. 6B). Knockdown of P65 by P65 siRNA inhibited Dox-promoted miR-34a-5p expression in H9c2 cells (Fig. 6C). In addition, inhibition of P53 by P53 siRNA could also suppress Dox-promoted miR-34a-5p expression (Fig. 6C). Collectively, our data suggest that up-regulation of miR-34a-5p in Dox-induced cardiomyocytes results from the activations of NF-κB and P53 signaling.

## Discussion

The *in vitro* ^23^ and *in vivo* ^24^ studies revealed the protective effect of DEX against anthracycline-induced cardiotoxicity. Iron may catalyse ROS formation in Dox-induced cardiotoxicity^4,5^, and DEX may chelate iron before it catalyses the conversion of O^2 •−^ and H_2_O_2_ to more damaging oxidants^25^. According to previous studies^26–28^, we established a rat model of cumulative cardiotoxicity, Dox and Dox combined with DEX were given for 4 or 8 weeks, respectively. Detections of plasma cTnT and Ca-Mg-ATP revealed that significant cardiotoxicity was achieved after 4-week and 8-week Dox treatment, but DEX could efficiently alleviate Dox-induced cardiac damage. MDA detection indicated that DEX treatment could also alleviate Dox-induced global redox injury in rats. Additionally, results of echocardiography assay and Masson staining demonstrated that significant and cumulative heart dysfunction and cardiac remodeling were achieved after 4-week and 8-week Dox treatment, but DEX could significantly attenuate Dox-induced cardiotoxicity.

Recently, some miRNAs, such as miR-146^29^, -34c^30^, -30^31^, -532-3p^32^ and let-7g^33^, have been reported involved in Dox-induced cardiotoxicity. Since significant cardiac cardiomyopathy was achieved in rats at either 4 or 8weeks after Dox treatment, we detected miRNA profiles in rat myocardium after 4-week Dox or Dox + DEX treatments in this study. As expected, some miRNAs were shown dysregulated in the myocardium and plasma of rats received 4-week Dox or Dox plus DEX treatments. Notablely, miR-34a-5p was observed consistently increased in the myocardium and plasma of 4-week Dox-treated rats, but was reversed in the myocardium and plasma of 4-week Dox plus DEX-treated rats, as well as in the myocardium and plasma of rats received 8-week Dox or Dox plus DEX treatments (data not shown). Additionally, hsa-miR-34a-5p was found significantly elevated in plasma of DLBCL patients received 9-week or 16-week epirubicin treatment. Therefore, miR-34a-5p was shown involved in Dox-induced cardiotoxicity, and it was also a potential biomarker for Dox-induced cardiotoxicity.

H9c2 cell line derives from heart tissue of embryonic BDIX rats. This kind of cell possesses features of skeletal muscle, including expressing nicotinic receptors and producing a muscle-specific creatine phosphokinase isoenzyme^34^. H9c2 cells were used to investigate the mechanism of Dox-induced cell death and apoptosis of cardiomyocytes^35^. In the present study, we observed that miR-34a-5p was markedly increased in the apoptotic H9c2 cells after Dox treatment, as well as in the supernatant. These data indicated that the elevated plasma miR-34a-5p of Dox-treated rats and of DLBCL patients received epirubicin treatment was derived from myocardium with Dox-induced cardiotoxicity.

Overexpression of miR-34a-5p increased the mitochondrial depolarization (Fig. 3E), Bax expression and the activated caspase-3, and attenuated Bcl-2 expression in H9c2 cells (Figs 3D,5C, Supplementary Figure 2). These data revealed that miR-34a-5p exerted the pro-apoptotic effect in Dox-treated cardiomyocytes, which was supported by previous reports^36,37^.

Sirt1, an NAD-dependent deacetylase, mediates deacetylation of targets such as PGC-1α, FOXO3, p53 and NF-κB, has profound effect on mitochondrial function, apoptosis and inflammation^38^. The present study has provided several lines of evidence to support the notion that miR-34a-5p enhances Dox-induced apoptosis through targeting Sirt1. First, the *in silico* prediction indicated that Sirt1 was a potential target of miR-34a-5p, and the dual luciferase assay revealed that miR34a-5p specifically bound to the 746–752, 1236–1242 sites in the 3′-UTR of Sirt1. Next, we detected the expression of Sirt1 in myocardium of rats received Dox or Dox plus DEX treatments. Results of RT-qPCR and Western blotting revealed that Sirt1 protein expression was significantly decreased in rat myocardium post-Dox treatment, but could be reversed by DEX intervention, without significant change of Sirt1 mRNA expression. In addition, we determined Sirt1 expression in H9c2 cells exposed to Dox treatment. Expression of Sirt1 at both mRNA and protein levels was significantly decreased in Dox-treated H9c2 cells. Next, H9c2 cells were separately transfected with scramble control and miR-34a-5p mimic for 24 hrs. Our data showed that Sirt1 protein expression was significantly reduced in miR-34a-5p-modified H9c2 cells, but Sirt1 mRNA level remained unchanged. The above results revealed that miR-34a inhibits Sirt1 expression at the post-transcriptional level. Furthermore, we separately transfected H9c2 cells with miR-34a-5p mimic and siRNA targeting Sirt1, followed by examining expressions of Bax and Bcl-2. Western blotting showed that Bax was increased, but Bcl-2 was down-regulated in H9c2 cells transfected with miR-34a-5p mimic or Sirt1 siRNA. Collectively, we demonstrated that miR-34a-5p participated in Dox-induced cardiotoxicity by targeting Sirt1. This conclusion has been supported by previous studies showing that Sirt1 was a target gene of miR-34a-5p^39–41^.

Moreover, our data showed that Bcl-2 protein, but not Bcl-2 mRNA, was significantly suppressed by miR-34a-5p (Figs 3D,5C), indicating that miR-34a-5p modulates Bcl-2 expression at the post-transcriptional level, which is also supported by the previous report that Bcl-2 was a target gene of miR-34a-5p^42^.

p66Shc, an isoform of the shcA adapter molecule, modulates intracellular redox balance by increasing ROS concentration as a critical mediator of intracellular oxidative signals transduction^43,44^. p66shc has been reported to mediate mitochondrial cell death pathways through increasing lipid peroxidation-induced apoptosis^22^. In the absence of p66shc, mice exhibited increased resistance to ethanol-induced mitochondrial ROS generation^45^. The p66Shc^−/−^ mouse was also reported as a model of prolonged lifespan associated with increased resistance to oxidative stress^46^. Moreover, p66Shc has been known negatively regulated by Sirt1 through deacetylation of histone H3 lysine 9 binding to the p66Shc promoter region^47^. Accordingly, our present data showed that expression p66shc was negatively with Sirt1 in Dox-induced cardiotoxicity *in vivo* and in *vitro*. As expected, overexpression of Sirt1 could suppress p66shc expression, and knockdown of Sirt1 could upregulated p66shc in H9c2 cells. Additionally, we also revealed that p66shc was positively correlated with Bax expression, but was negatively correlated with Bcl-2 expression in Dox-induced cardiotoxicity *in vivo* and in *vitro* (Fig. 5). This conclusion has been supported by previous study showing that deletion of p66Shc protected mouse splenocytes from cell death induced by hypoxia and resulted in increased Bcl-xL and decreased Bax expression^48^. Therefore, activation of Sirt1/p66shc pathway mediated the pro-apoptotic effect of miR-34a-5p on cardiomyocytes in Dox-induced cardiotoxocity.

NF-κB signaling has been shown to participate in Dox-induced cardiomyocyte apoptosis^49,50^. Our present study has confirmed that the NF-κB/p65 signaling pathway was activated in Dox-treated H9c2 cells. We used NF-κB P65 siRNA, NF-κB P65 inhibitor JSH23 and QNZ, and P53 siRNA to further verify the participations of NF-κB P65 and P53 in Dox-promoted upregulation of miR-34a-5p in H9c2 cells. The present study also showed that Dox-upregulated miR-34a-5p was decreased after enforced expression of Sirt1 in H9c2 cells, this result has been supported by the negative feedback loop between miR-34a-5p, SIRT1 and p53^39,40^.

**Figure 7:**
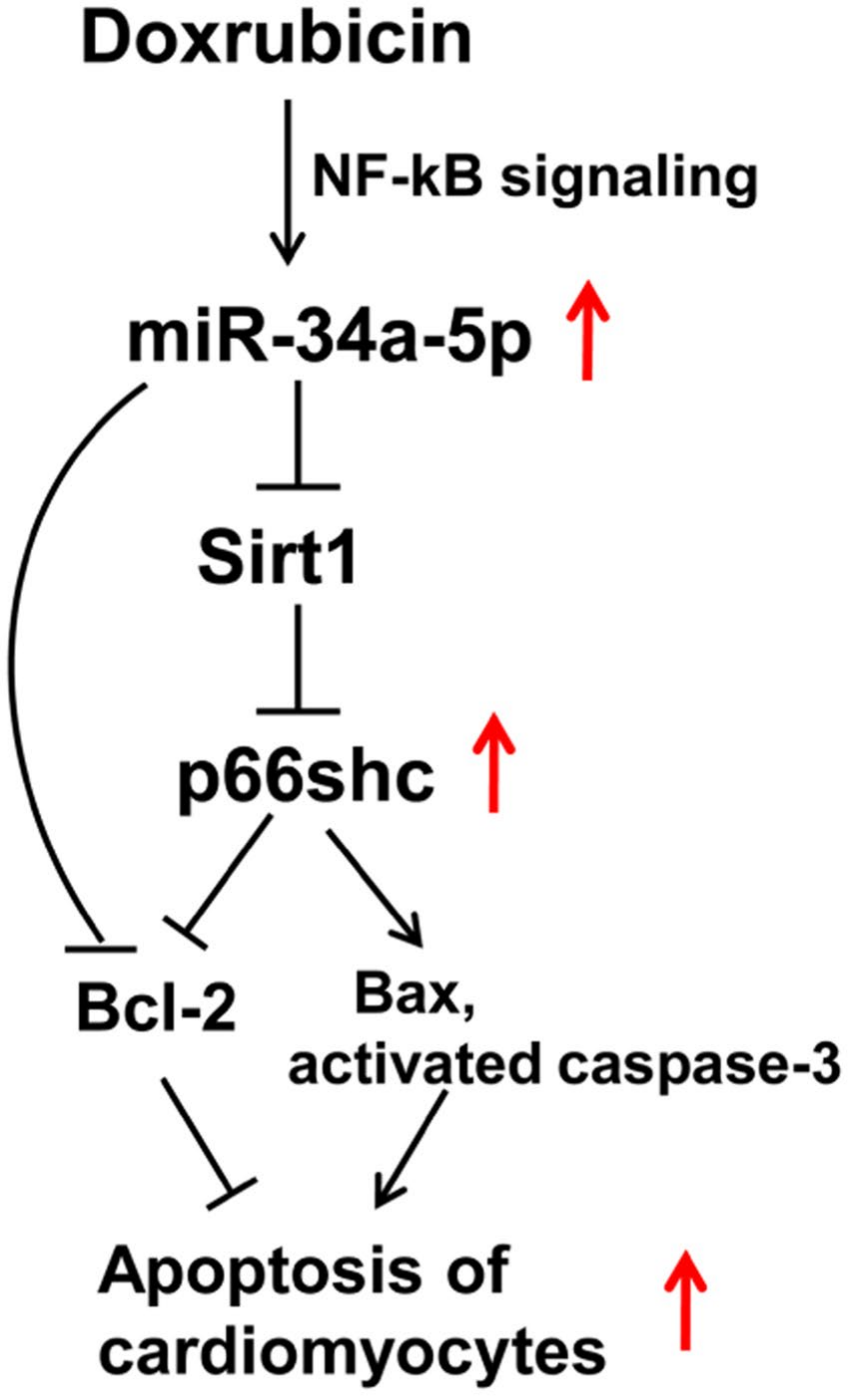
Schematic diagram of the mechanism whereby miR-34a-5p exerts the pro-apoptotic effect in DOx-induced toxic cardiomyopathy. MiR-34a-5p is upregulated in Dox-induced H9c2 cells via NF-κB pathway. MiR-34a-5p enhances p66shc expression by targeting Sirt1, resulting in increases of Bax and the activated caspase-3, and a decrease of Bcl-2 (Bcl-2 is also a direct target of miR-34a-5p), and contributing to Dox-induced apoptosis of cardiomyocytes.

Taken together, our results have demonstrated that miR-34a-5p was up-regulated in myocardium and plasma of rats with Dox-induced cardiotoxicity, and DEX could suppress the Dox-promoted upregulation of miR-34a-5p. MiR-34a-5p could enhance the apoptosis of cardiomyocytes by increasing Bax expression and the activated caspase-3, and inhibiting Bcl-2 expression. Sirt1 was confirmed as a target of miR-34a-5p, and Sirt1/p66shc pathway mediated the pro-apoptotic effect of miR-34a-5p on cardiomyocytes. We also concluded that activation of NF-κB signaling pathway mediates the upregulation of miR-34a-5p in Dox-induced cardiomyocytes. Therefore, the present study suggests that miR-34a-5p might be a potential target for therapy and diagnosis of Dox-induced cardiotoxicity (as shown in Fig. 7).

## Electronic supplementary material

Supplementary Information
