# Supplementary material for: Activation of miR-34a-5p/Sirt1/p66shc pathway contributes to doxorubicin-induced cardiotoxicity
Source: Sci Rep. 2017 Sep 19;7:11879. doi: 10.1038/s41598-017-12192-y (PMC5605522; doi:10.1038/s41598-017-12192-y)
Supplement: Supplementary file 1 — Supplementary Information [file 41598_2017_12192_MOESM1_ESM.pdf]

Activation of miR-34a-5p/Sirt1/p66shc pathway contributes to doxorubicin-induced cardiotoxicity

Jie-Ning Zhu[1,2]*, Yong-Heng Fu[1,2]*,Zhi-qin Hu[1,2]*, Wen-Yu li[3], Chun-Mei Tang[1,2], Hong-Wen Fei[1], Hui Yang[1,2], Qiu-xiong Lin[1,2], De-Ming Gou[4], Shu-Lin Wu[1,2], Zhi-Xin Shan[1,2]

[1] Guangdong Cardiovascular Institute, Guangdong Provincial Key Laboratory of Clinical Pharmacology, Guangzhou 510080, China; [2] Research Center of Medical Sciences, Guangdong General Hospital, Guangdong Academy of Medical Sciences, Guangzhou 510080, China; [3]Lymphoma Division, Cancer Center, Guangdong General Hospital, Guangdong Academy of Medical Sciences, Guangzhou, 510080, China; [4]College of Life Science, Shenzhen University, Shenzhen, Guangdong, 518060, China.

* These authors contributed equally to this work.

Correspondence to: Zhi-Xin Shan, e-mail: zhishan@aliyun.com

Supplementary Data

Table 1 Primers used in real-time qRT-PCR

Gene	Sequence (5′- 3′)	Product size（bp）
Sirt1	F, TACCCCATGAAGTGCCTCAA	195
	R, CCTTTTGTGTTCGTGGAGGT	
Bcl-2	F, GGGAGATCGTGATGAAGTACA	218
	R, GCTGAGCGCAGGCCCAG	
BAX	F, CCAGCTCTGAACAGATCATG	201
	R, CAATCATCCTCTGCAGCTCC	
NF-κB P65	F, ACCATCATCACGCTGGAAGA	202
	R, TCTTTTGCTGGGGAGAGGAG	
P53	F, GGACAGCTTTGAGGTTCGTG	202
	R, CCCACGGATCTTAAGGGTGA	
GAPDH	F, CAAGAAGGTGGTGAAGCAGG	200
	R, CCACCCTGTTGCTGTAGCC	

Table.2 Assessment of the cardiac function by echocardiography

Group	Control	Dox-4w	Dox+DEX-4w	Dox-8w	Dox+DEX-8w
LVAWd	2.04±0.14	1.76±0.27	1.85±0.06	1.52±0.29**	1.91±0.12$^{\triangle}$
LVAWs	2.96±0.17	2.39±0.43*	2.49±0.18	2.06±0.39***	2.60±0.19$^{\triangle}$
LVIDd (mm)	5.53±0.44	7.66±0.62***	7.07±0.90	7.63±0.74***	7.21±0.82
LVIDs (mm)	3.31±0.48	5.52±0.35***	4.74±0.53$^{\#}$	6.02±0.61***	4.81±0.37$^{\triangle\triangle}$
LVPWd	2.07±0.21	1.85±0.23	2.00±0.30	1.63±0.31*	1.87±0.14
LVPWs	2.90±0.27	2.51±0.24*	2.45±0.35	2.06±0.47**	2.61±0.28$^{\triangle}$

LVAWd, left ventricular anterior wall end-diastolic thickness, LVAWs, the systolic left ventricular anterior wall thickness, LVIDd, left ventricular internal dimension at end-diastole, LVIDs, left ventricular internal dimension at end-systole, LVPWd, the left ventricular posterior wall end-diastolic thickness, LVPWs, the left ventricular posterior wall end-systolic thickness, EF, ejection fraction. FS, fraction shrinkage. Data represent the mean±SD, $^{*}p<0.05$, $^{**}p<0.01$, $^{***}p<0.001$ vs. control group, $^{\#}p<0.05$, $^{\#\#}p<0.01$, $^{\#\#\#}p<0.001$ vs. Dox-4w group, $^{\triangle}p<0.05$, $^{\triangle\triangle}p<0.01$, $^{\triangle\triangle\triangle}p<0.001$ vs. Dox-8w group.

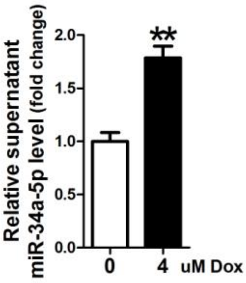

Figure 1. Determination of miR-34a-5p level in the supernatant of Dox-treated H9c2 cells. Level of miR-34a-5p in the supernatant of H9c2 cells exposed to 4 uM Dox was detected by RT-qPCR assay. Data are shown as mean ± sem. $^{**}p < 0.01$ *vs* 0 uM Dox control. $n = 3$.

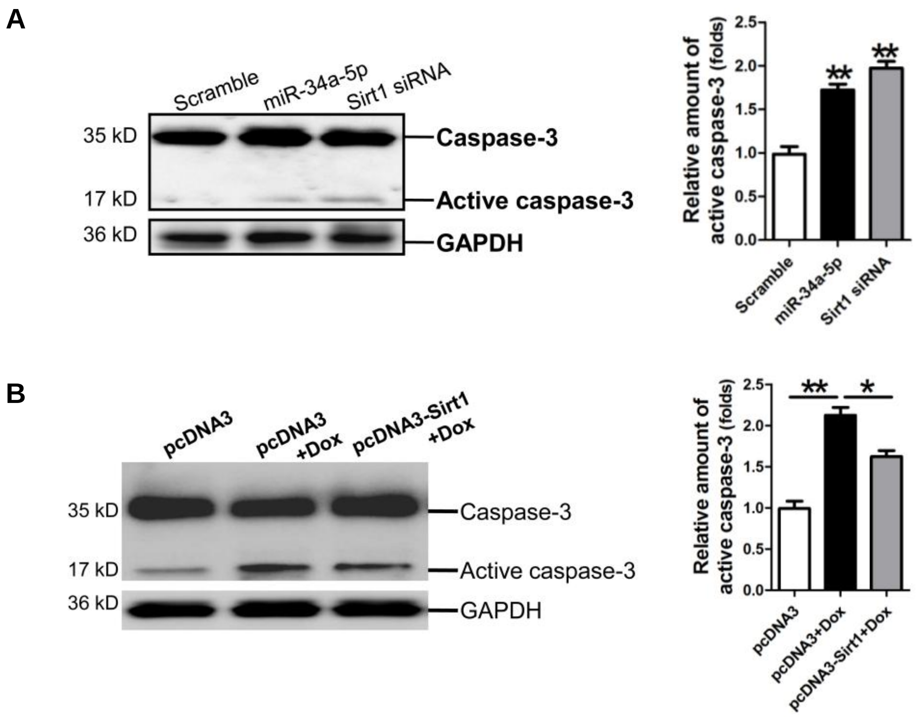

Figure 2. Determination of Caspase-3 protein level in H9C2 by Western blot assay. (A) H9c2 cells were transfected with miR-34a-5p mimic and Sirt1 siRNA, respectively. Data are shown as mean ± sem. $^{**}p < 0.01$ *vs* scramble group. $n = 3$. (B) H9c2 cells were treated by 4 uM Dox after transfection with pcDNA3-Sirt1. Data are shown as mean ± sem. $^{*}p < 0.05$, $^{**}p < 0.01$. $n = 3$.
